# Large health disparities in cardiovascular death in men and women, by ethnicity and socioeconomic status in an urban based population cohort

**DOI:** 10.1016/j.eclinm.2021.101120

**Published:** 2021-08-29

**Authors:** Janet M. Kist, Gideon W.G. Smit, Albert T.A. Mairuhu, Jeroen N. Struijs, Rimke C. Vos, Petra G. van Peet, Hedwig M.M. Vos, Edith D. Beishuizen, Yvo W.J. Sijpkens, Rolf H.H. Groenwold, Mattijs E. Numans

**Affiliations:** a Department of Public Health & Primary Care, Leiden University Medical Center, Campus The Hague, the Netherlands; b Department of Internal Medicine, HAGA Teaching Hospital, The Hague, the Netherlands; c National Institute for Public Health and the Environment, Bilthoven, the Netherlands; d Department Internal Medicine, HMC Hospital, The Hague, the Netherlands; e Department of Clinical Epidemiology, Leiden University Medical Center, Leiden, the Netherlands; f Department of Biomedical Data Science, Leiden University Medical Center, Leiden, the Netherlands

**Keywords:** Cardiovascular death, ethnicity, socioeconomic status, health disparities, health equity

## Abstract

**Background:**

Socioeconomic status and ethnicity are not incorporated as predictors in country-level cardiovascular risk charts on mainland Europe. The aim of this study was to quantify the sex-specific cardiovascular death rates stratified by ethnicity and socioeconomic factors in an urban population in a universal healthcare system.

**Methods:**

Age-standardized death rates (ASDR) were estimated in a dynamic population, aged 45–75 in the city of The Hague, the Netherlands, over the period 2007–2018, using data of Statistics Netherlands. Results were stratified by sex, ethnicity (country of birth) and socioeconomic status (prosperity) and compared with a European cut-off for high-risk countries (ASDR men 225/100,000 and women 175/100,000).

**Findings:**

In total, 3073 CVD deaths occurred during 1·76 million person years follow-up. Estimated ASDRs (selected countries of birth) ranged from 126 (95%CI 89–174) in Moroccan men to 379 (95%CI 272–518) in Antillean men, and from 86 (95%CI 50–138) in Moroccan women to 170 (95%CI 142–202) in Surinamese women. ASDRs in the highest and lowest prosperity quintiles were 94 (95%CI 90–98) and 343 (95%CI 334–351) for men, and 43 (95%CI 41–46) and 140 (95%CI 135–145), for women, respectively.

**Interpretation:**

In a diverse urban population, large health disparities in cardiovascular ASDRs exists across ethnic and socioeconomic subgroups. Identifying these high-risk subgroups followed by targeted preventive efforts, might provide a basis for improving cardiovascular health equity within communities. Instead of classifying countries as high-risk or low-risk, a shift towards focusing on these subgroups within countries might be needed.

**Funding:**

Leiden University Medical Center and Leiden University

**Evidence before this study**

 

There is mounting evidence that socioeconomic status and ethnicity are separate risk elevating predictors for cardiovascular death, alongside the traditional cardiovascular risk factors such as, smoking, age, blood pressure, sex and cholesterol. On mainland Europe, socioeconomic status and ethnicity are not incorporated as predictors in country-level risk charts. No guidelines or studies were found regarding regional or within country CVD risk assessments or adjustment for socioeconomic and ethnic subgroups.

In preparation for this manuscript Pubmed was searched until May 2021 for "cardiovascular mortality", "cardiovascular prevention", "cardiovascular prediction", "socioeconomic status", "disposable household income", "low income", "ethnicity", "health disparity" and "health inequalities".

**Added value of this study**

This study showed that even in a city/region within a country with a universal healthcare system, the differences in CVD deaths between socioeconomic and ethnic subgroups are substantial. Subgroups in our urban population (low socioeconomic, Surinamese and Antillean men and women) showed high standardized CVD death rates, comparable to CVD death rates of high-risk countries.

**Implications of all the available evidence**

Socioeconomic status and ethnicity are known risk factors for cardiovascular mortality. As long as these factors are not incorporated as predictors in country-level risk charts in mainland Europe, a European age-standardized death rates cut-off for a within country implementation of cardiovascular low-risk or high-risk charts in subgroups might be needed. Also, knowledge on specific subgroups in regions with higher cardiovascular death, may provide a basis for regional tailoring of cardiovascular risk management and prevention measures aimed at improving health equity.

Alt-text: Unlabelled box

## 1 Introduction

Cardiovascular diseases are the number one cause of mortality, accounting for 15 million cardiovascular disease (CVD) deaths worldwide [1]. In Europe, 45% of all mortality is caused by CVD (men 40 and women 49%) [2]. The primary prevention of CVD in Europe is guided by public health policies and the 2016 European Society of Cardiology (ESC) *Guidelines on cardiovascular prevention in clinical practice*, in which countries are recommended to implement the use of the high-risk or low-risk SCORE chart based on their WHO Global Health Observatory country level cardiovascular age-standardized death rate (ASDR) [3,4]. The (arbitrarily chosen) cut-off level to distinguish high-risk and low-risk countries is an ASDR of 225 and 175 per 100,000 persons for men and women per year, respectively (45–75 years of age) [3,5]. For example, the Netherlands, France, and Belgium are advised to use the chart for low-risk countries, whereas Poland, Croatia, and the Czech Republic are examples of high-risk countries. The high-risk chart has lower treatment thresholds. For an individual with the same sex, age, smoking status, cholesterol, blood pressure measurement, and comparable risk modifying factors as a positive family history of CVD, this could amount to a 5–10 year earlier advised start of antihypertensive or cholesterol-lowering medication [3].

Apart from the traditional risk factors such as age, sex, blood pressure, smoking, cholesterol and chronic conditions as diabetes that are commonly used for prediction of cardiovascular risk, ethnicity and socioeconomic status also play a role [3,6]. However, currently these relevant influencing factors, are mentioned in the guidelines, but are not incorporated as predictors in the risk chart that are in use in (mainland) Europe today [3]. As far as we know, only QRISK incorporates both ethnicity and socioeconomic status as predictors in their cardiovascular risk scores for use in the UK [7].

Previous studies showed that the incidence of CVD deaths among specific ethnic subgroups differs from the general population and also differs within ethnic groups between European regions [3,8]. With regard to ethnic subgroups a European study on ethnicity by country of birth (individuals 35–74 years old, 1996–2006) in six European countries showed higher cardiovascular mortality rate ratios in the Netherlands in Caribbean (Surinamese and Antillean) and Turkish subgroups compared to the Dutch subgroup [8].

In a lifetime perspective CVD death is higher in women than in men in Europe (coronary heart disease, heart failure and stroke), whereas in younger age groups CVD death is higher in men [3]. The overall higher cardiovascular mortality in women is believed to be related to under-recognition, under-diagnosing and under-treatment of women's CVD [9].

Furthermore, many studies showed a strong inverse relation between socioeconomic status and CVD deaths. Already in 1961, Marmot et al. described that cardiovascular mortality was more common among working class men and women compared to the middle and upper class in England and Wales [10]. Recent reviews from 2017 and 2018 described similar relations between CVD and socioeconomic status [11,12]. In addition, a meta-analysis from 2017 in 1.7 million European, US and Australian adults, found a clear association between low socioeconomic status and premature CVD death, also after adjusting for traditional risk factors (in men as well as in women) [6]. Possible explanations for these associations are genetic predispositions for the development of risk factors such as hypertension and diabetes, lifestyle behaviors, and environmental influences such as pollution, stress and varying access to amenities in neighbourhoods [12,13].

Since the ESC classification in high-risk and low-risk countries does not account for sex-specific heterogeneity in ethnicity and socioeconomic status within countries, the use of country level high-risk or low-risk charts might be inappropriate for high-risk subgroups in regions and cities within countries. Therefore, the aim of this study was to gain more insight in the ASDR for cardiovascular mortality in different sex-specific ethnic and socioeconomic subgroups in a highly urbanized area in the Netherlands, by comparing cardiovascular ASDR (ICD10 I00-I99) in subgroups to the ESC cut-off for high-risk and low-risk countries.

## 2 Methods

### 2.1 Setting

This study was situated in the city of The Hague in the Netherlands, an urban area of 100 km^2^ with around half a million inhabitants. In The Hague the proportion of citizens with a non-western ethnicity is 35%, compared to 12% in the average population in the Netherlands [14]. Also, the number of citizens with minimal income levels (22%) is higher than the national average (14%) [14]. The Dutch health system has a universal health care system which is widely recognized for its equal access to healthcare [15].

### 2.2 Study design

The study design was a dynamic population cohort design. Individuals aged 45 to 75 years registered as residents in the study area between 2007 and 2018 were included in the analyzes. The age group was chosen in accordance with the age groups used in the *2016 ESC Guideline on cardiovascular disease prevention* cut-off for high-risk countries [3]. Person years at risk for CVD deaths were calculated.

### 2.3 Data collection

Data was individually linked on cardiovascular mortality, age, country of birth (derived from Dutch population register) and socioeconomic status (derived from Dutch tax register) from Statistics Netherlands [16]. More information on the linking procedure can be found elsewhere [17].

### 2.4 Ethnicity by country of birth

Ethnicity of individuals was based on the classification available at Statistics Netherlands and was defined by the country of birth of that person or by country of birth of the mother. If the mother was Dutch, ethnicity was defined by the country of birth of the non-Dutch father [18]. Country of birth as a proxy of ethnicity is common in Dutch literature [18]. Data on self-perceived or other classifications of ethnicity was not available [19,20]. Data on ethnicity was analyzed because of the research aim, to gain more insight in CVD death in regional ethnic subgroups. For the current study, we analyzed the ethnic subgroups of The Hague, which where large enough to meet ASDR model requirements (a minimum of 20–25 CVD deaths per stratified group): Dutch, Surinamese, Moroccan, Indonesian, Turkish, Antillean, and German. The other countries of birth were combined to one group (subdivision by continent in Appendix 3). Ethnicity data was available from 2007 to 2018 and had no missing data.

### 2.5 Socioeconomic status

For this study, socioeconomic status was measured by prosperity level. This is composed of two elements, first the amount of standardized disposable household income and second household wealth. The standardized disposable household income represents “the net amount a household can spend on an annual basis, adjusted for any differences in household size and composition” [17]. Household wealth equates to the total value of assets minus the value of outstanding liabilities. Assets broadly include things such as property and savings, whilst liabilities are financial debts [17]. As the prosperity data is incomplete in the year of death, we have individually used the previous year's prosperity level for all participants. The combined prosperity level provided by Statistics Netherlands was measured in percentiles of the population as a whole, and categorized in quintiles. Method of ranking, exacting boundaries and collinearity of disposable household income and prosperity on ASDRs are specified in Appendix 1. From 2011 onward, the prosperity levels were available for most citizens, thus confining the time period for prosperity analyzes to 2012–2018.

Furthermore, concerning prosperity analyzes students and individuals living in an institution were censored (2%), and 3% was classified missing (and were excluded from the analyzes). For the largest ethnic groups, i.e., Dutch and Surinamese, ASDRs were also calculated in quintiles of prosperity. The other ethnics groups were not large enough to allow ASDR estimation in quintiles of prosperity [21]. Other individual proxies for socioeconomic status such as highest level of education attained and occupation were not available [12].

### 2.6 Cardiovascular mortality

The main outcome of this study was CVD death, which was based on ICD-10 death diagnoses I00-I99. ICD-10 death codes were available in the Dutch Death Registry (part of Statistics Netherlands), which was found reliable with more than 98% known causes of death [22].

## 3 Analysis

Cardiovascular ASDRs based on the WHO World population were estimated stratified by subgroups (sex, ethnicity and/or prosperity level) [23]. The ASDR is a descriptive statistic and is an incidence rate, used for comparing death rates of different (sub)populations. According to the WHO definition, the “age-standardized mortality rate is a weighted average of the age-specific mortality rates per 100,000 persons, where the weights are the proportions of individuals in the corresponding age groups of the WHO standard population” [24]. The ASDR can be interpreted as the projected number of CVD deaths that would have occurred per year if the population was of the same age distribution as the WHO standard population [23]. Our estimates of ASDRs were based on data from our eleven years’ study period and compared with the ESC defined cut-off for high-risk and low-risk countries of 225/100,000 for men and 175/100,000 for women.

To compare the ASDRs of the ethnic and prosperity male and female subgroups reciprocally we also estimated standardized rate ratios (SRR). The SRR is a relative rate ratio, used to compare differences between death rates, and is calculated by dividing the standardized death rate of a subgroup by the sex-specific standardized death rate of the (chosen) reference population [21]. If the SRR is 1.6 for a certain subgroup, there are 1.6 times or 60% more standardized CVD deaths in that subgroup compared to the reference population. We chose the Dutch subgroup as the sex-specific reference population for the ethnicity subgroups, and the 3^rd^ quintile of prosperity of the overall population as the reference for prosperity subgroups-analyzes (3rd quintile because this central quintile contains the average income).

Data was analyzed using *R* statistics and the packages Epitools (ageadjust direct), version 0.5–10 and DSR (directly standardized rate ratios) version 0.2.2 [25,26]. JK, GS and RG had access to the data in 2019 (preliminary research) and JK and RG in 2020.

## 4 Sensitivity analyzes

First, in ethnicity subgroups we compared atherosclerotic cardiovascular ASDRs (ICD 10 diagnoses used in the SCORE prediction models) with overall cardiovascular ASDRs (ICD10 I00–I99), since overall cardiovascular ASDRs also contains non-atherosclerotic diagnoses such as thrombosis (Appendix 2). Second, since CVD deaths from remaining (not selected) countries of birth were too small to be analyzed by individual country, these were analyzed as one group “other countries”. To assess global differences within this combined group, ASDRs by continent were estimated (Appendix 3). Last, deaths of Dutch citizens occurring abroad are by default labelled as “R99, unknown cause of death”. Considering the possibility that these could have been CVD deaths, data on unknown cause of death were aggregated and compared with CVD deaths by ethnic subgroup. (Appendix 4).

## 5 Medical ethical considerations

The ethical committee of the area (LUMC Leiden) waived the need for ethical approval (number G18.070).

## 6 Role of funding source

The funding source had no role in study design, data analysis, interpretation and writing of the manuscript.

## 7 Results

### 7.1 Characteristics of the study population

The population we included was 160,000 individuals on average per year, with 50% men/women. The study population accounts for the total 45–75 year old registered inhabitants of the region during the study period from 2007 to 2018. In men 2062 CVD deaths occurred in 879,000 person years and in women 1011 CVD deaths occurred in 876,000 person years (Table 1). The average follow-up time was 6·7 years. Albeit marginally, the total CVD deaths per year declined more steeply in men (from 304 per 100,000 person years in 2007 to 174 in 2017) than in women (160 to 100).

**Table 1:** Characteristics of the 45–75 years old multi-ethnic urban cohort in the Netherlands, 2007–2018.

	Men				Women			
Ethnicity	n CVD deaths	PY	Median Age	(25th - 75th Percentile)	n CVD deaths	PY	Median Age	(25th - 75th Percentile)
Dutch	1298	527000	57.8	(51.2-64.8)	643	524000	58.6	(51.7-65.8)
Surinamese	247	78000	54.6	(49.5-61.3)	136	92000	55.0	(49.7-61.6)
Turkish	61	40000	52.3	(48.2-59.0)	21	34000	53.0	(48.6-60.0)
Moroccan	37	32000	54.5	(49.0-62.8)	18	25000	54.5	(49.3-61.4)
Indonesian	109	50000	57.5	(51.0-64.2)	62	51000	57.6	(51.3-64.6)
Antilleans	44	14000	54.3	(49.2-60.9)	15	15000	54.8	(49.6-61.2)
Germans	74	17000	63.6	(55.6-69.1)	44	18000	64.9	(57.7-70.0)
other countries	192	121000	53.1	(48.5-59.3)	72	117000	53.5	(48.8-60.0)
**Total**	**2062**	**879000**	**56.3**	**(50.2-63.6)**	**1011**	**876000**	**57.0**	**(50.6-64.5)**
Prosperity Quintiles								
1st (low prosperity)	317	93000	56.0	(50.1-63.5)	155	101000	57.6	(51.0-64.9)
2nd	230	80000	57.7	(50.5-66.6)	112	89000	59.0	(51.2-67.3)
3rd	157	86000	56.0	(50.0-64.1)	75	86000	56.6	(50.3-64.5)
4th	118	94000	55.8	(50.1-62.9)	51	89000	55.9	(50.3-63.0)
5th (high prosperity)	128	126000	57.9	(51.4-64.9)	55	117000	57.7	(51.3-64.8)
**Total**	**950**	**479000**			**448**	**482000**		
Dutch by Prosperity								
1st (low prosperity)	166	33000	58.1	(51.5-64.7)	76	38000	60.1	(52.8-66.7)
2nd	148	42000	61.9	(53.0-68.6)	83	50000	63.4	(54.0-69.2)
3rd	111	52000	57.6	(50.8-65.6)	57	53000	58.5	(51.3-65.9)
4th	72	64000	56.4	(50.5-63.6)	31	60000	56.6	(50.6-63.8)
5th (high prosperity)	88	94000	58.4	(51.8-65.2)	34	83000	58.3	(51.8-65.3)
**Total**	**585**	**285000**			**281**	**284000**		
Surinamese by Prosperity								
1st (low prosperity)	48	10000	56.8	(50.8-64.1)	31	15000	58.5	(52.0-66.0)
2nd	ud	ud	55.1	(49.4-63.0)	ud	ud	55.1	(49.6-62.0)
3rd	ud	ud	54.0	(49.3-60.7)	ud	ud	54.0	(49.2-60.0)
4th	ud	ud	54.8	(49.9-60.9)	ud	ud	54.1	(49.7-59.7)
5th (high prosperity)	ud	ud	56.2	(50.8-62.4)	ud	ud	55.4	(50.7-61.1)
2nd - 5th	72	34000			26	39000		
**Total**	**120**	**44000**			**57**	**54000**		

Legend Table 1. Characteristics of the 45–75 years old multi-ethnic urban cohort in the Netherlands, 2007-2018.

CVD deaths, cardiovascular deaths ICD-10 diagnoses I00-I99.

PY, person years at risk.

Etnicity, According to country of birth (person or parent).

Prosperity, Disposable household income combined with household wealth, in quintiles of the population. Based on prosperity level in the previous calendar year. Data about time interval 2012–2018.

ud, undisclosed, due to low numbers.

For ethnicity, Germans and Antilleans (both 2% of total) were the smallest subgroups and Dutch was the largest subgroup (60% of total). Oldest subgroups were the Germans and Dutch, and youngest were Moroccan subgroups, see Table 1 for median ages and Appendix 5 for age distribution within ethnic subgroups. Appendix 6 presents the distribution of the WHO standard population.

### 7.2 CVD deaths total population and ethnicity

**Fig. 1:**
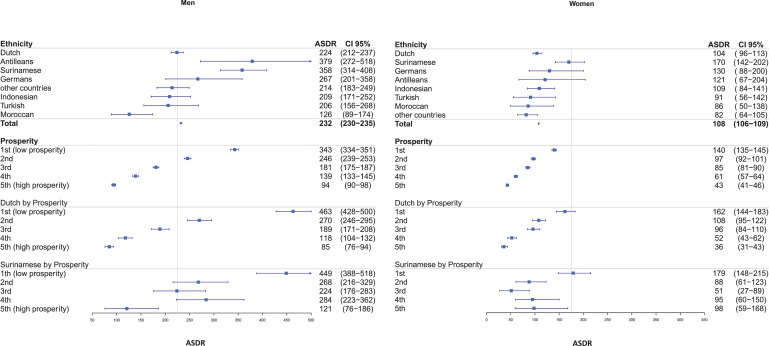
Standardized cardiovascular death rates among 45–75 years old ethnic and socioeconomic subgroups in The Netherlands, compared to a European cut-off.

The ASDR for the overall population of The Hague was 232 (per 100,000 person years, 95% CI 230–235) for men and 108 (95% CI 106-109) for women (Fig. 1). With regard to ethnicity, Moroccan men had the lowest ASDR of 126 (95% CI 89–174). Two ethnic groups had an ASDR above the high-risk country ESC cut-off, namely Antillean men with an ASDR of 379 (95% CI 272–518) and Surinamese men with an ASDR of 358 (95% CI 314–408). In women, ASDRs were all below the high-risk country ESC cut-off and ranged from 82 (95% CI 64–105) in “other countries” to 170 (95% CI 142–202) in Surinamese.

### 7.3 CVD deaths and prosperity levels

ASDRs for prosperity showed an inverse relation between prosperity and CVD deaths. In men ASDR ranged from 94 (per 100,000 person years, 95% CI 90-98) to 343 (95% CI 334-351) (Fig. 1). In the two lowest quintile groups for prosperity ASDRs were above the men's cut-off of 225. In women, ASDRs ranged from 43 (95% CI 41-46) to 140 (95% CI 135-145), all below the women's cut-off point of 175.

In the two largest subgroups (Dutch and Surinamese) the poorest prosperity groups showed approximately equal ASDRs. ASDR of ethnicity by prosperity level showed the highest ASDR of all analyzed groups: In the Dutch subgroup by prosperity levels, ASDRs ranged from 85 (per 100,000 person years, 95% CI 76–94) to 463 (95% CI 428–500) in men, and 36 (95% CI 31–43) to 162 (95% CI 144–183) in women (Fig. 1). Both Dutch men and women showed an inverse relationship between CVD deaths and prosperity. For Surinamese, by prosperity levels, ASDRs ranged in men from 121 (95% CI 76–186) to 449 (95% CI 388–518), and in women the range was 98 (95% CI 144–183) to 179 (95% CI 148–215).

### 7.4 Standardized rate ratio (SRR)

For ethnicity, comparing men and women ethnic subgroups to the Dutch men and women reference population with estimation of SRRs, Surinamese (1·6 with 95% CI 1·55-1·65; *p* < 0·0001) and Antillean men (1·69 with 95% CI 1·59-1·79; *p* < 0·0001) as well as Surinamese women (1·63 with 95% CI 1·56-1·69; *p* < 0·0001) showed a 60–69% higher standardized CVD deaths in these subgroups (Table 2).

**Table 2:** Standardized cardiovascular death rate ratios in a 45–75 years old multi-ethnic cohort in The Netherlands.

	Men			Women		
	SRR	95% CI	p value	SRR	95% CI	p value
Dutch	**1.00**	(ref)		**1.00**	(ref)	
Antilleans	**1.69**	(1.59-1.79)	<0.0001	**1.16**	(0.98-1.33)	0.06
Surinamese	**1.60**	(1.55-1.65)	<0.0001	**1.63**	(1.56-1.69)	<0.0001
Germans	**1.19**	(1.10-1.28)	<0.0001	**1.25**	(1.13-1.37)	<0.0001
other countries	**0.95**	(0.90-1.01)	0.08	**0.71**	(0.61-0.81)	<0.0001
Indonesian	**0.93**	(0.87-1.00)	0.04	**1.05**	(0.96-1.13)	0.24
Turkish	**0.92**	(0.83-1.01)	0.10	**0.87**	(0.72-1.02)	0.12
Moroccan	**0.56**	(0.45-0.67)	<0.0001	**0.82**	(0.66-0.98)	0.05
**Prosperity**						
1st (low prosperity)	**1.89**	(1.81-1.98)	<0.0001	**1.65**	(1.53-1.76)	<0.0001
2nd	**1.36**	(1.27-1.45)	<0.0001	**1.13**	(1.01-1.25)	0.03
3rd	**1.00**	(ref)			**1.00**	(ref)
4th	**0.77**	(0.66-0.88)	<0.0001	**0.71**	(0.56-0.86)	0.002
5th (high prosperity)	**0.52**	(0.42-0.63)	<0.0001	**0.51**	(0.36-0.65)	<0.0001
**Dutch by Prosperity**						
1st (low prosperity)	**2.56**	(2.47-2.65)	<0.0001	**1.90**	(1.77-2.04)	<0.0001
2nd	**1.49**	(1.39-1.59)	<0.0001	**1.26**	(1.12-1.40)	<0.0001
3rd	**1.04**	(0.94-1.14)	0.43	**1.13**	(0.99-1.27)	0.05
4th	**0.65**	(0.54-0.77)	<0.0001	**0.61**	(0.44-0.78)	<0.0001
5th (high prosperity)	**0.47**	(0.36-0.57)	<0.0001	**0.43**	(0.26-0.59)	<0.0001
**Surinamese by Prosperity**						
1th (low prosperity)	**2.48**	(2.35-2.61)	<0.0001	**2.10**	(1.95-2.25)	<0.0001
2nd	**1.48**	(1.30-1.66)	<0.0001	**1.03**	(0.78-1.29)	0.82
3rd	**1.24**	(1.04-1.43)	0.01	**0.60**	(0.18-1.01)	0.25
4th	**1.57**	(1.37-1.77)	<0.0001	**1.12**	(0.80-1.43)	0.44
5th (high prosperity)	**0.67**	(0.32-1.01)	0.17	**1.15**	(0.81-1.49)	0.37

SRR, standardized mortality rate ratio.

Ref, Reference population (Dutch for ethnicity groups, 3rd quintile of overall prosperity for prosperity groups).

CVD deaths, cardiovascular deaths ICD-10 diagnoses I00-I99.

Ethnicity, According to country of birth (person or parent).

Prosperity, Disposable household income combined with household wealth, in quintiles of the population.

Looking at SRRs in prosperity subgroups, the inverse relation between prosperity and cardiovascular mortality is clearly visible in all prosperity subgroups. In the overall population, SRR in prosperity, compared to the 3rd quintile, showed in men a 48 lower to 89% higher standardized death rates and in women 49 lower to 65% higher (SRR men 0·52 with 95%CI 0·42–0·63; *p* < 0·0001 to 1·89 with 95%CI 1·81–1·98; *p* < 0·0001and women 0·51 with 95% CI 0·36–0·65; *p* < 0·0001 to 1·65 with 95% CI 1·53-1·76; *p* < 0·0001). In the Dutch by prosperity subgroups, these differences were higher, in the lowest prosperity quintile men had 156% and women had 90% higher standardized death rates, compared to the reference population (3rd quintile of overall prosperity; *p* < 0·0001). Also, in the Surinamese by prosperity subgroups, we found that in the lowest prosperity quintile standardized CVD deaths in men were 148% higher and in women 110% higher compared to the 3^rd^ quintile of overall prosperity (*p* < 0·0001) (Table 2).

### 7.5 Sensitivity analyzes

In the sensitivity analysis using atherosclerotic CVD death as an endpoint instead of overall CVD death, analogous ethnicity subgroups (Surinamese men and women and Antillean men) showed elevated ASDRs and rate ratios (Appendix 2). Second, when analyzing “other countries” by continent, the “European other” category was the largest, and Oceania the smallest. African men showed the highest ASDR (234 95% CI 153–364) and “Asian other” women the lowest (41 95% CI 17–85) (Appendix 4). Last, mortality due to unknown cause (ICD10 R99), which could have been CVD deaths, was especially high in Moroccan and Turkish subgroups (Appendix 5).

## 8 Discussion

Large differences in CVD death rates were observed between sex-specific ethnic subgroups and socioeconomic subgroups in the city of The Hague. The highest CVD death rates were found in Antillean (men), Surinamese (men and women) and low-prosperity subgroups (men and women, irrespective of ethnic background). At the same time high prosperity subgroups notably showed relatively low death rates. Furthermore, combined analyzes of ethnicity and prosperity in Surinamese and Dutch displayed equally high death rates for the lowest prosperity groups. The death rates of certain subgroups of men (Surinamese, Antillean, low prosperity) were found to be much higher than what was to be expected of a European low-risk country. At the same time, CVD death rate ratios (a relative measure) exhibited analogous differences between ethnic and socioeconomic sex-specific subgroups.

In this study in The Hague, Surinamese and Antillean subgroups showed cardiovascular mortality rate ratio ranging from 1·16–1·69, compared to the Dutch subgroup. A study in the Netherlands (part of a European study), showed a somewhat lower (averaged) cardiovascular mortality rate ratio in 35–75 years old of 1·16 in the Caribbean subgroup (Surinamese and Antillean subgroups), compared to the Dutch subgroup [8]. Possible explanations for variations in results could be differences in age distribution, unmeasured relevant determinants that play a role in a highly urbanized context, or heterogeneity in group composition (for example, the Surinamese in The Hague in 2008 consisted of 76% South Asians compared to 45% in the general Surinamese subgroup in the Netherlands) [25].

The observed inverse relation between lower socioeconomic status and higher CVD death rates in men and women is consistent with literature [6,11]. Several intermixing factors have been correlated to higher CVD death in low socioeconomic subgroups. These include, higher prevalence of traditional risk factors (smoking, elevated blood pressure, higher cholesterol levels), less healthy lifestyle behavior, higher stress levels and unhealthy neighborhood conditions in low socioeconomic areas (for example, the density of fast-food take away outlets is higher in low socioeconomic areas, and a larger distance to fast-food take away outlets was ‘associated with a slightly higher dietary quality’ in these areas of The Hague) [27]. Also, under-treatment of cardiovascular events contributed to a higher incidence of CVD deaths in lower socioeconomic subgroups, among others due to differences in medical interventions or treatment adherence [6,12]. Besides the traditional risk factors for CVD death (i.e., diabetes, smoking, age, hypertension, blood pressure) ethnicity and socioeconomic status are independently associated with CVD death [3,6]. The health inequity in the incidence of CVD deaths for ethnic and socioeconomic subgroups as shown in this study in a city in a (low-risk) country with a universal health system, is evident. The intermixing associated factors on social, health and environmental levels leading to CVD health inequity, necessitate a multilevel approach [3].

The finding of higher CVD death rates in men compared to women (45–75 years old) is also consistent with results of previous studies [3,9]. In a lifetime perspective CVD deaths are higher in women [3,9]. Alternative risk prediction models for women are considered in studies and literature, such as lifetime or adjusted CVD risk models for women [3,9]. Using a (relative) sex-specific comparison as was done in this regional study, the high-risk subgroups in men and women (Surinamese, Antillean and low socioeconomic men and women) can be identified to guide for example multilevel preventive measures in a collaborative community and health care network in regions or communities.

Previous studies on regional differences in CVD deaths largely describe between-country differences. One study comparing within-country mortality (ischaemic hearth disease and cerebrovascular disease) showed differences in mortality in the United Kingdom, Portugal, Poland and Finland [28]. Studies on CVD deaths within smaller regions or cities by ethnicity and socioeconomic status were not found. Comparing our city's highest (low socioeconomic Surinamese and Dutch) and lowest ASDR (high socioeconomic) to Europe's country level ASDRs, the ASDRs in men in our region range from high-risk countries as Romania and Albania, to ASDRs in men and women even lower than France (which has the lowest ASDRs of Europe) [5]. This illustrates the large cardiovascular health inequity within our region.

The completeness of the datasets used, particularly regarding ethnicity and prosperity, is a strength of this study. Another strength is the duration of follow-up, merging information on eleven years, which allowed us to analyse CVD deaths in various subgroups.

This study has also had limitations. First, we used country of birth as a proxy for ethnicity. Self-perceived ethnicity could have led to different subgroups and results. Second, although combining information across eleven years of follow-up allowed for detailed subgroup analyzes, sample size was still insufficient to investigate time trends and interactions (e.g., between ethnicity and socioeconomic status). Third, causes of death may be misclassified possibly leading to an underreporting of CVD death. Notably, for example when a Dutch citizen dies in Morocco, this is classified as death of unknown cause. Particularly among Moroccan and Turkish subgroups, the percentage of deaths with unknown cause was large. If these were CVD deaths, the standardized mortality rates in these groups may actually be larger than what we were able to report in this study (Appendix 4). Fourth, socioeconomic status was analyzed using a combined income and wealth measure. CVD deaths can be differently associated with various socioeconomic measures (education, profession, income, wealth and neighborhood). Income and wealth are both considered as valid indicators to use in relation to CVD, but other measures could have led to different results [11]. Last, methods of direct standardization level out age differences to a certain extent. When a subgroup is relatively much older in age distribution compared to other subgroups (as the Germans in our study), estimation of ASDR can be higher (age bias).

While the Netherlands is a country with a low risk of CVD death according to ESC classification, several subgroups in the highly-urbanized region of The Hague show CVD death rates which on a country level would have had to lead to the use of the high-risk score chart. Using CVD death rates to divide populations in regions or cities in high-risk and low-risk subgroups, and combining this information with targeted regional community and health care cardiovascular prevention plans, might be part of a feasible solution towards the improvement of cardiovascular health equity. This merits future research.

The current ESC guideline advises adjustment of the risk-estimation for certain ethnic subgroups [3]. The Dutch guideline recommends earlier screening in these subgroups, but still the same low-risk chart is being used [29]. A European cut-off for high-risk and low-risk countries could be amended to a within country distinction between high-risk and low-risk subgroups. As long as there is no adequate sex-specific risk prediction model adjusted for ethnicity and socioeconomic status, populations within countries might benefit from a shift from a European cut-off between countries to a within country cut-off for high-risk and low-risk subgroups, specified by research based on risk benefit studies, tailored on sex, ethnicity and socioeconomic subgroups.

Our current study showed large health disparities in CVD death rates in ethnic and socioeconomic subgroups in a diverse urban population. In addition to country level risk charts, identifying high-risk subgroups in regions using death rates followed by targeted preventive efforts, might provide a basis for improving cardiovascular health equity within communities. Instead of classifying European countries as high- or low-risk, a shift towards focusing on these subgroups within countries might be needed.

## Author contributions

JK, MN, RG, JS, RV, PP, GS, AM, HV, EB and YS contributed to the conception or design of the work, or to the acquisition, analysis, or interpretation of data for the work. The underlying data was verified by JK, GS and RG. JK drafted the manuscript. MN, RG, JS, RV, PP, GS, AM, HV, EB and YS critically revised the manuscript. All gave final approval and agree to be accountable for all aspects of work ensuring integrity and accuracy

## Data sharing statement

The data used for this study is part of a larger study on health equity in the city of The Hague (*ELAN Happy and Healthy The Ha*gue, sub study *Vascular The Hague*). Access to the research dataset used in this study is restricted to organizations eligible for access to Statistics Netherlands [16]. For these organizations access to this research dataset, is possible after application and approval of a sound research proposal by the LUMC department of Public Health and Primary Health Care (https://www.lumc.nl/org/elan/).

The research protocol is available via the author (J.M.Kist@LUMC.nl).

## Declaration of Competing Interest

None of the authors reported a conflict of interest.

## Appendix Supplementary materials

Image, application 1
