# Supplementary material for: Large health disparities in cardiovascular death in men and women, by ethnicity and socioeconomic status in an urban based population cohort
Source: eClinicalMedicine. 2021 Aug 29;40:101120. doi: 10.1016/j.eclinm.2021.101120 (PMC8408518; doi:10.1016/j.eclinm.2021.101120)
Supplement: Supplementary file 1 [file mmc1.docx]

**Appendix**

**Appendix 1. Prosperity level, disposable household income and wealth (boundaries and comparison)**

To determine the percentile ranking of household prosperity, individuals are ranked from high to low according to their household disposable income and wealth. The ranking position is then summed up and divided by two. For individuals displaying negative household wealth (for example due to a mortgage) combined with a high household income, a correction is then made, and these individuals are subsequently ranked higher. The combined ranking is then recalculated into percentiles.

Because of this method’s exacting boundaries (and re-ranking), prosperity quintiles cannot be specified in euros. However, the quintile boundaries for disposable household income and household wealth are established and identified.^1^

**Appendix 1a. Quintile boundaries of disposable household income and wealth**

|  |  | **Disposable Household Income (x 1000 euro)** | | | |  | **Wealth (x 1000 euro)** | |  |  |
| --- | --- | --- | --- | --- | --- | --- | --- | --- | --- | --- |
| **Year** |  | **1st-2nd** | **2nd-3rd** | **3rd-4th** | **4th-5th** |  | **1st-2nd** | **2nd-3rd** | **3rd-4th** | **4th-5th** |
| 2012 |  | 15·6 | 20·5 | 25·8 | 33·2 |  | -0·4 | 8·1 | 68·2 | 225·6 |
| 2013 |  | 15·6 | 20·6 | 26 | 33·6 |  | -3·8 | 3·9 | 50·8 | 199·4 |
| 2014 |  | 16 | 21·2 | 26·8 | 34·7 |  | -4 | 3·7 | 50·3 | 199·1 |
| 2015 |  | 16·3 | 21·4 | 27·3 | 35·3 |  | -1·9 | 5·6 | 54·7 | 206·6 |
| 2016 |  | 16·8 | 22·2 | 28·4 | 36·6 |  | -0·7 | 6·9 | 58·9 | 214 |
| 2017 |  | 17·2 | 22·7 | 29 | 37·4 |  | 0 | 10·1 | 70·7 | 231·4 |

Source: Statistics Netherlands

**Appendix 1b. Standardized cardiovascular death rates of disposable household income compared to prosperity among 45-75 years old men and women in The Hague (2012-2018)**


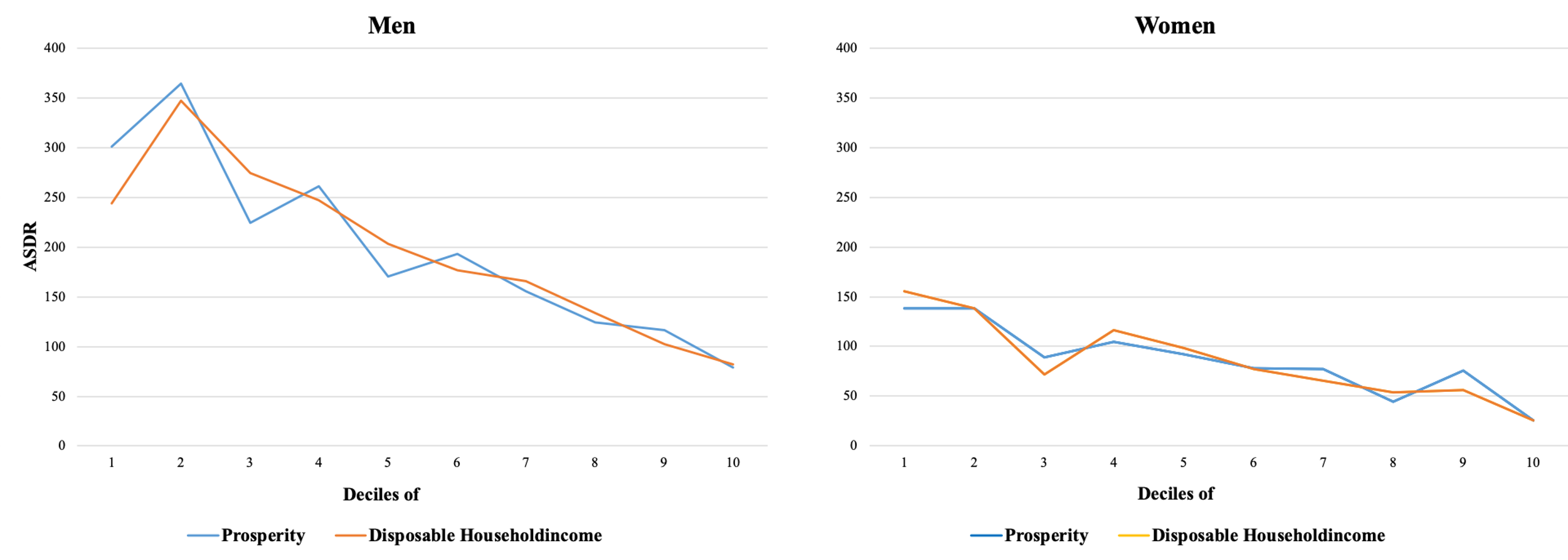


Collinearity in CVD death (ASDR’s) per decile of prosperity and disposable income

ASDR, Age standardized death rate (/100,000 person-years at risk), standardized to the World population, population 45-75 years, based on CVD death diagnoses (I00-I99)

Deciles, 1^st^ is the lowest decile, 10^th^ is the highest decile of prosperity and disposable household income

Prosperity, standardized disposable household income combined with household wealth.

Disposable income, the net amount a household can spend on an annual basis, adjusted for any differences in household size and composition

**Appendix 2. Standardized atherosclerotic cardiovascular deaths in a 45-75 years old multi-ethnic cohort in the Netherlands (2007-2018)**

| Atherosclerotic cardiovascular deaths, ICD-10 diagnoses I10-I25, R96, I46, I47-I51, I61-I65 (except I62.0), G45, I67-I69 (except I67.1), I70-I72 |
| --- |
| ASDR, age standardized death rate (/100,000 person-years at risk), standardization to the World population, period 2007-2018 |
| lci-uci, lower and upper 95% confidence interval ASDR |
| Ethnicity, according to country of birth (person or parent) |
| SRR, standardized mortality rate ratio |
| lci SRR - uci SRR, lower and upper 95% confidence interval SRR |
| Ref, Reference population (Dutch for ethnicity groups) |

**Appendix 3. Cardiovascular death rates and characteristics in a 45-75 years old cohort in the Netherlands, combination category “other countries” subdivided by continent (2007-2018)**

| **Men** |  |  |  |  |  |  |  |  |  |  |
| --- | --- | --- | --- | --- | --- | --- | --- | --- | --- | --- |
| **Continent** | **median** | **25th** | **75th** | **py** | **crude** | **ASDR** | **lci** | **uci** |  |  |
| Africa | 51·5 | 48·1 | 56·1 | 24000 | 173 | 236 | 153 | 364 |  |  |
| America other | 53·2 | 48·7 | 59·4 | 9000 | 47 | 95 | 21 | 281 |  |  |
| Asia other | 54·0 | 49·1 | 60·6 | 32000 | 157 | 222 | 163 | 296 |  |  |
| Australia/Oceania | 52·1 | 48·2 | 57·4 | 1000 | 111 | 207 | 5 | 1430 |  |  |
| Europe other | 53·2 | 48·5 | 60·0 | 56000 | 170 | 211 | 169 | 261 |  |  |
|  |  |  |  |  |  |  |  |  |  |  |
| **Women** |  |  |  |  |  |  |  |  |  |  |
| **Continent** | **median** | **25th** | **75th** | **py** | **crude** | **ASDR** | **lci** | **uci** |  |  |
| Africa | 51·6 | 48·0 | 56·7 | 13000 | 94 | 120 | 55 | 261 |  |  |
| America other | 53·6 | 49·0 | 59·4 | 17000 | 46 | 76 | 30 | 166 |  |  |
| Asia other | 53·8 | 49·0 | 60·0 | 28000 | 28 | 41 | 17 | 85 |  |  |
| Australia/Oceania | 52·8 | 48·6 | 58·3 | 1000 | ud | ud | ud | ud |  |  |
| Europe other | 53·8 | 48·9 | 60·8 | 58000 | 76 | 94 | 68 | 127 |  |  |
|  |  |  |  |  |  |  |  |  |  |  |
| CVD death, ICD 10 diagnoses I00-I99 | | |  |  |  |  |  |  |  |  |
| Continent, ethnicity/country of birth (person or parent) per continent | | | | | |  |  |  |  |  |
| median, median of age (years) | |  |  |  |  |  |  |  |  |  |
| 25th - 75th, 25th and 75th percentile age | | |  |  |  |  |  |  |  |  |
| py, person years at risk | |  |  |  |  |  |  |  |  |  |
| crude, crude CVD death rate/100.000 py | | |  |  |  |  |  |  |  |  |
| ASDR, age standardized death rate (/100,000 person-years at risk), standardization to the World population, period ‘07- ‘18 | | | | | | | | | | |
| lci - uci, lower and upper 95% confidence interval ASDR | | | | |  |  |  |  |  |  |
| ud, undisclosed due to low numbers | | |  |  |  |  |  |  |  |  |

**Appendix 4. Deaths due to unknown cause (R99) and CVD deaths, specified by ethnicity, The Hague, population 45-75 years of age, between 2007-2018**

|  | **Male** |  |  | **Female** |  |
| --- | --- | --- | --- | --- | --- |
| **Ethnicity** | **R99 deaths** | **CVD deaths** |  | **R99 deaths** | **CVD deaths** |
| Dutch | 158 | 1298 |  | 62 | 643 |
| Surinamese | 64 | 247 |  | 25 | 136 |
| Turkish | 54 | 61 |  | 29 | 21 |
| Moroccan | 35 | 37 |  | 15 | 18 |
| Indonesian | 22 | 109 |  | ud | 62 |
| Antileans | 10 | 44 |  | ud | 15 |
| Germans | ud | 74 |  | ud | 44 |
| other countries | 60 | 192 |  | 46 | 72 |
| **Total** | **403** | **2062** |  | **177** | **1011** |

R99 deaths, death due to unknown cause (ICD 10 R99, among other deaths in a non-Dutch country are coded as R99)

ud, the total number of R99 death is smaller than 10 and is therefore undisclosed from Statistics Netherlands

CVD deaths, ICD 10 diagnoses I00-I99

**Appendix 5. Age distribution ethnicity**

|  | **Male (%)** |  |  |  |  |  |  |
| --- | --- | --- | --- | --- | --- | --- | --- |
| **Age** | **Dutch** | **Surinamese** | **Turkish** | **Moroccan** | **Indonesian** | **Antilleans** | **Germans** |
| [45,50) | 20 | 27 | 36 | 30 | 21 | 28 | 13 |
| [50,55) | 19 | 24 | 26 | 22 | 20 | 24 | 11 |
| [55,60) | 18 | 19 | 16 | 15 | 19 | 19 | 13 |
| [60,65) | 18 | 15 | 10 | 13 | 18 | 14 | 20 |
| [65,70) | 14 | 9 | 7 | 12 | 13 | 9 | 23 |
| [70,75) | 10 | 6 | 5 | 8 | 9 | 5 | 21 |
|  |  |  |  |  |  |  |  |
|  | **Female (%)** |  |  |  |  |  |  |
| **Age** | **Dutch** | **Surinamese** | **Turkish** | **Moroccan** | **Indonesian** | **Antilleans** | **Germans** |
| [45,50) | 19 | 26 | 34 | 29 | 20 | 27 | 10 |
| [50,55) | 18 | 24 | 25 | 23 | 20 | 24 | 9 |
| [55,60) | 18 | 19 | 16 | 19 | 19 | 20 | 12 |
| [60,65) | 18 | 14 | 12 | 13 | 17 | 14 | 20 |
| [65,70) | 15 | 10 | 8 | 9 | 13 | 9 | 25 |
| [70,75) | 12 | 6 | 5 | 7 | 10 | 6 | 25 |

%, percentage of total

**Appendix 6. Distribution of the WHO Standard population, weights for ASDR calculations**

| **Age** | **WHO weight** | **Weight world 45-75 yrs old (%)** |
| --- | --- | --- |
| 45-50 | 6·04 | 24 |
| 50-55 | 5·37 | 22 |
| 55-60 | 4·55 | 18 |
| 60-65 | 3·72 | 15 |
| 65-70 | 2·96 | 12 |
| 70-75 | 2·21 | 9 |
|  | 24·85 | 100 |

WHO weight: distribution of the WHO standard population

Weight world 45-75 yrs old, weight of the world recalculated for ages 45-75 years old.
